# Occurrence of Thermophilic Microorganisms in Different Full Scale Biogas Plants

**DOI:** 10.3390/ijms21010283

**Published:** 2019-12-31

**Authors:** Ivan Kushkevych, Jiří Cejnar, Monika Vítězová, Tomáš Vítěz, Dani Dordević, Yannick J. Bomble

**Affiliations:** 1 Department of Experimental Biology, Faculty of Science, Masaryk University, 62500 Brno, Czech Republic; 451355@mail.muni.cz (J.C.); vitezova@sci.muni.cz (M.V.); 2 Department of Agricultural, Food and Environmental Engineering, Faculty of AgriSciences, Mendel University, 61300 Brno, Czech Republic; tomas.vitez@mendelu.cz; 3 Department of Plant Origin Foodstuffs Hygiene and Technology, Faculty of Veterinary Hygiene and Ecology, University of Veterinary and Pharmaceutical Sciences, 61242 Brno, Czech Republic; dani_dordevic@yahoo.com; 4 Bioscience Center, National Renewable Energy Laboratory, 16253 Denver West Parkway, Golden, CO 80401, USA; Yannick.Bomble@nrel.gov

**Keywords:** renewable energy, biogas, Illumina sequencing, thermophilic microorganisms, anaerobic digesters

## Abstract

Background: In recent years, various substrates have been tested to increase the sustainable production of biomethane. The effect of these substrates on methanogenesis has been investigated mainly in small volume fermenters and were, for the most part, focused on studying the diversity of mesophilic microorganisms. However, studies of thermophilic communities in large scale operating mesophilic biogas plants do not yet exist. Methods: Microbiological, biochemical, biophysical methods, and statistical analysis were used to track thermophilic communities in mesophilic anaerobic digesters. Results: The diversity of the main thermophile genera in eight biogas plants located in the Czech Republic using different input substrates was investigated. In total, 19 thermophilic genera were detected after 16S rRNA gene sequencing. The highest percentage (40.8%) of thermophiles was found in the Modřice biogas plant where the input substrate was primary sludge and biological sludge (50/50, w/w %). The smallest percentage (1.87%) of thermophiles was found in the Čejč biogas plant with the input substrate being maize silage and liquid pig manure (80/20, w/w %). Conclusions: The composition of the anaerobic consortia in anaerobic digesters is an important factor for the biogas plant operator. The present study can help characterizing the impact of input feeds on the composition of microbial communities in these plants.

## 1. Introduction

Anaerobic digestion is one of the technologies that can process and reduce biodegradable waste, thus limiting its environmental impact. Anaerobic processes are effective tools to reduce pollution and they fully meet cleaner energy production objectives. It has been used for wastewater treatment and more recently, for processing biodegradable industrial and agricultural wastes [1,2,3,4,5]. Anaerobic processes occur naturally in wetlands, swamps, and in the digestive tracts of ruminants [6]. Anaerobic microorganisms were also discovered in landfills where they degrade biodegradable municipal waste. The product of anaerobic metabolism is biogas [7,8], which is classified as a source of renewable energy [9]. During the anaerobic process, biochemical degradation of organic polymers to methane (CH_4_) and carbon dioxide (CO_2_) occurs [10,11,12,13]. The major components of biogas are CH_4_ (55% vol–70% vol) and CO_2_ (30% vol–45% vol) [14,15,16]. Anaerobic microorganisms are responsible for establishing a stable environment at different stages of biogas production [7,8,12].

Thermophilic microorganisms live at high temperatures 50–122 °C. Most of them belong to the domain of *Archaea* [17]. This group of microorganisms can be classified, according to their optimal growth temperatures, as thermophiles (50–64 °C), extremophiles (65–79 °C) and hyperthermophiles (≥80 °C) [18]. Thermophilic microorganisms are often isolated from waste water discharge, bio waste streams, acid mine effluents as well as geothermal and volcanic areas, terrestrial hot springs, submarine hydrothermal vents, geothermally heated oil reserves and oil wells, sun-heated litter, and soils/sediments, throughout the world [19].

There are not many information about thermophilic microorganisms included in methane production and oxidation processes. It was found that mushroom compost piles contain 2 × 10^8^ thermophilic methanogens per gram dry matter. The processes involved in methane oxidation are important since 90% of methane reaching atmosphere is in oxidized form [4].

The advances of thermophilic conditions in anaerobic digestion are represented by the fact that gas is formed within shorter amount of time than when anaerobic digestion is done under mesophilic conditions. Though, higher energy is necessary for the maintenance of thermophilic conditions in biogas plant reactor. The heating in digesters that are operating at mesophilic and thermophilic levels results in effective denaturation of weed seeds and also pathogens reduction. The pathogen reduction, after 20 days, is almost 100%, meaning that thermophilic temperatures are of crucial importance for pathogens elimination. Other biogas plant digesters, operating at room temperatures, have much lower elimination level of pathogens [6].

It should be noted that thermophilic microorganisms may be involved in the process of methanogenesis, but their diversity in mesophilic biogas plants is still not well characterized, especially with respect to the effect of substrate variation. Additionally, the changes in the distribution of thermophiles in mesophilic conditions has never been well studied either. On the one hand, it appears that these microorganisms would not be able to grow under mesophilic conditions (40–50 °C) as their growth would be too slow and unable to compete with mesophiles, but on the other hand, this hypothesis has never been studied in this context. Indeed, there is a lack of information about the metabolic activity of these microorganisms under mesophilic conditions and there remains a possibility that they could be involved in the process of methanogenesis. The prevalence of thermophilic populations in mesophilic biogas plants and their composition with respect to different substrate ratios has rarely been studied. This study aims at reducing this knowledge gap.

## 2. Results

**Figure 1:**
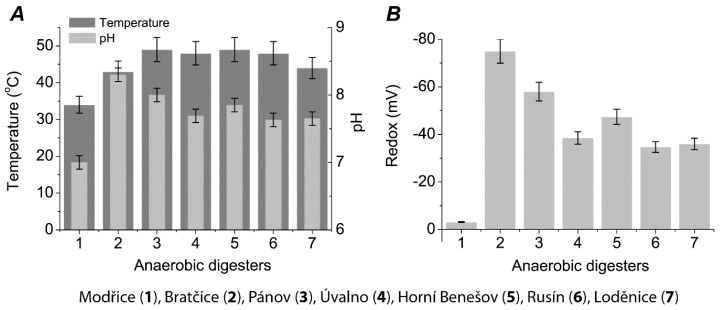
Physical characteristics of anaerobic digesters considered in this study (M ± SE, *n* = 3): temperature and pH (**A**), redox (**B**).

**Figure 2:**
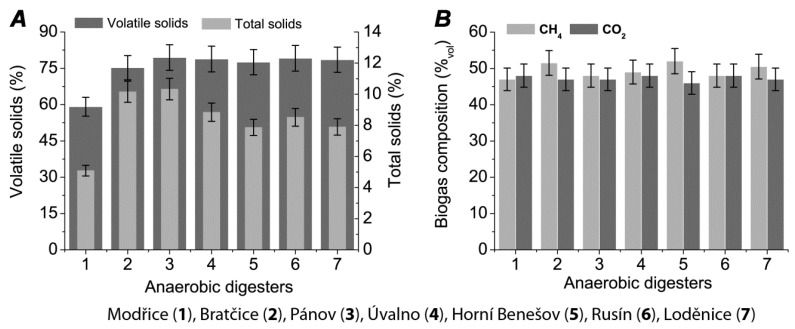
Amount of solids (**A**) and biogas production (**B**) (M ± SE, *n* = 3).

**Figure 3:**
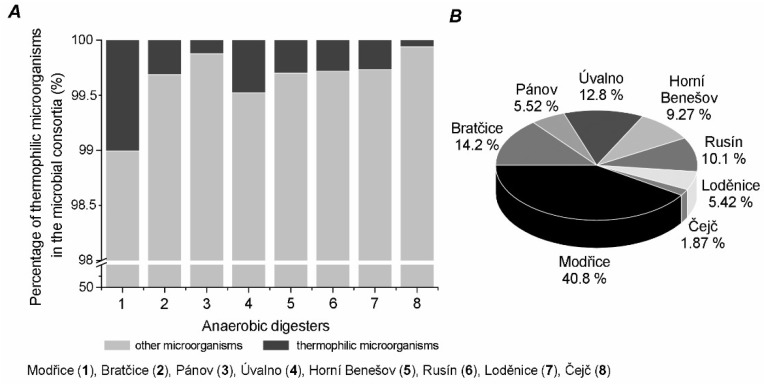
Percentage of thermophiles in the microbial community for each reactor (**A**) and percentage of thermophiles observed in all anaerobic fermenters together (**B**).

Seven operating biogas plants in the Czech Republic, each using different feeds, were selected as sources of microbial consortia for further analysis. At the present time, there are working 450 agricultural biogas plants in the Czech Republic. Most of them operating at mesophilic temperature (in the vicinity of 42 °C) to minimize high heat loss during winter time due to the low ambient temperatures. In this study, we selected plants using different operating conditions and ratios of input material. The physical and chemical properties (including: temperature, pH, oxidation-reduction potential (ORP), total volatile solids, and gasses: CH_4_, CO_2_, and H_2_) of these bioreactors are shown in Figure 1, Figure 2 and Figure 3. The data indicate that the highest temperature was measured in the Pánov reactor (49 °C), the lowest pH in the Modřice reactor (pH:7), and lowest ORP in the Bratčice reactor (−75 mV) (Figure 1).

Total and volatile solids in the different anaerobic digesters are shown in Figure 2A and are, for the most part, fairly similar with the exception of the Modřice reactor where total and volatile solids were significantly lower. The composition of the respective biogas in these reactors (for the two most dominant compounds, methane (CH_4_) and carbon dioxide (CO_2_)) is shown in Figure 2B. The levels of methane and CO_2_ produced ranged from 47% (Modřice) to 52% (Horní Benešov) and approximately 47% for all bioreactors. Levels of hydrogen were also detected and were significantly lower in the Úvalno and Loděnice bioreactor (0.0035%). The maximum level of hydrogen (0.0060%) was measured in the fermenter located in Horní Benešov. The percentage of other gases detected was in the range of 1.49% in Bratčice and 4.99% in Modřice.

We also studied the diversity of thermophilic microorganisms in these mesophilic biogas plants and evaluated the proportion of thermophiles in the microbial consortia. In all anaerobic digesters considered in this study, the proportion of thermophiles ranged from 0.06 to 1% and the composition of thermophiles was dependent on the composition of the input substrate in each biogas plant (Figure 3A). The most widespread genus among all biogas plants was *Syntrophaceticus* sp. and it was found in each fermenter and dominated in five of them. On the other hand, the lowest percentage and diversity of thermophilic genera (*Thermogymnomonas* and *Syntrophaceticus*) was observed in the Čejč fermenter.

**Figure 4:**
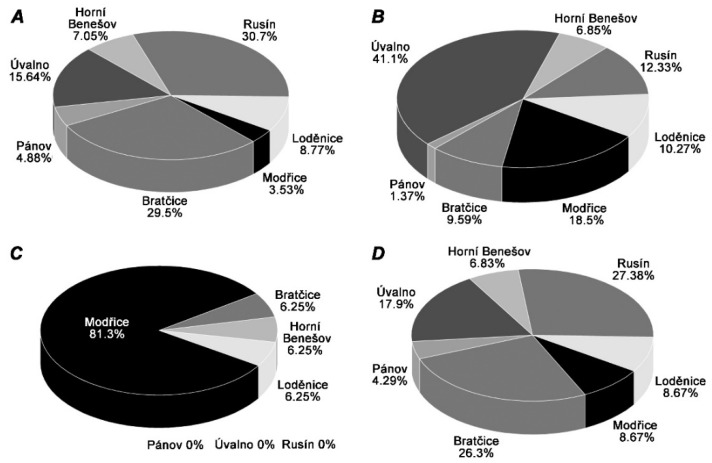
The most widespread genera of methanogenic microorganisms in the anaerobic digesters: *Methanoculleus* (**A**), *Thermogymnomonas* (**B**), *Methanobacterium* (**C**), total number (**D**).

The greatest diversity of thermophilic microorganisms was detected in the fermenter located in Modřice (Figure 3B), probably because this anaerobic fermenter is operating in a wastewater treatment plant. The sample from Modřice contained 11 different genera of thermophiles: *Thermogymnomonas* (6.5%), *Thermoflavimicrobium* (31%), *Thermovirga* (24%), *Thermoleophilum* (0.24%), *Thermanaeromonas* (0.24%), *Thermomonas* (2%), *Syntrophaceticus* (0.97%), *Fervidobacterium* (31%), *Kosmotoga* (3.6%), *Caldimicrobium* (0.24%), and *Oceanotoga* (0.48%). However, the dominant genera in this reactor were *Thermoflavimicrobium* (31.40%) and *Fervidobacterium* (30.67%). It should be noted that the *Thermoflavimicrobium* genus was also observed in the fermenter located in Horní Benešov but in very low abundance (1%). The *Fervidobacterium* genus was also detected in Rusín (0.98%) and Bratčice (2%) but in low abundance as well. The following methanogenic microorganisms were found in the bioreactors (Figure 4): *Methanoculleus*, *Thermogymnomonas*, and *Methanobacterium*. Bioreactors in Rusín and Bratčice had mostly *Methanoculleus* genus (30.7% and 29.5%, respectively), Úvalno *Thermogymnomonas* genus (41.1%), and Modřice *Methanobacterium* genus (81.3%). The following methanogenic genera were also detected: *Thermoplasmata*, *Methanospirillum*, *Thermoprotei*, *Methanobrevibacter*, *Methanolinea*, *Methanosaeta*, *Methanimicrococcus*, though not in significant amounts.

Within all biogas plants, the second highest diversity of thermophiles was found in Bratčice where there were 10 genera of thermophiles including *Syntrophaceticus* (38.24%), *Gelria* (23.53%), *Thermogymnomonas* (17.65%), *Oceanotoga* (10.78%), *Petrotoga* (4.90%), and 0.98% of other genera including *Desulfovirgula*, *Fervidobacterium*, *Moorella*, *Thermoactinomyces*, and *Thermosynthropa*. The presence of *Desulfovirgula* and *Thermosynthropa* genera were determined only in the fermenter where maize silage/sugar beet pulp (70/30, w/w%) was used as a substrate. The Bratčice fermenter showed a different microbial profile with the following genera: *Syntrophaceticus* (57.63%), *Gelria* (15.97%), *Oceanotoga* (11.11%), *Thermogymnomonas* (9.72%), *Fervidobacterium* (2.08%), *Thermoactinomyces* (1.38%), and 0.69% of the remaining genera being *Thermanaeromonas*, *Thermovirga*, *Petrotoga*. The composition of the microbial consortium was fairly similar in the Pánov fermenter, most likely because both fermenters process poultry litter. However, the microbial diversity was higher in Bratčice which can be the result of different ratios of input substrates, which are maize silage/whole crop silage/poultry litter (63/31/6, w/w%) in Bratčice and maize silage and poultry litter (92/8, w/w%) in Pánov.

**Figure 5:**
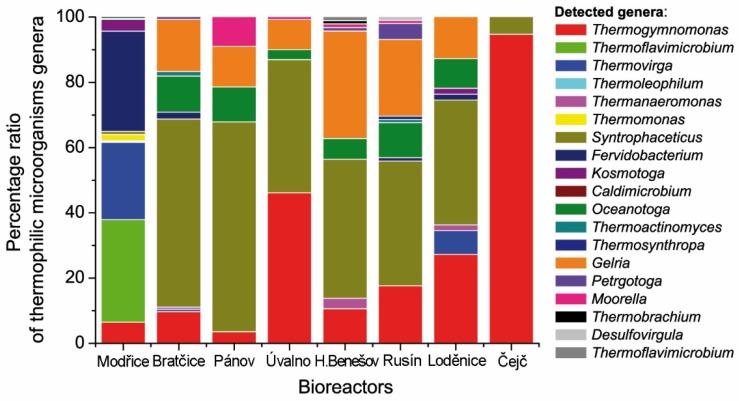
Percentage of each thermophilic genus in the overall population of thermophiles in each anaerobic fermenter.

Fermenters in Úvalno and Pánov had a similar diversity of thermophiles, even though substrate heterogeneity was higher in Úvalno where sugar beet pulp, maize silage, cattle manure, whole crop silage are used compared to maize silage and poultry litter in Pánov. The lower diversity and abundance of thermophiles were detected in the Čejč fermenter (Figure 5). There were only two dominant genera, *Syntrophaceticus* and *Thermogymnonas*, in ratios of 5.27% and 94.73%, respectively. In this fermenter, maize silage and liquid pig manure were used. The Loděnice and Rusín fermenters process the same substrate, sugar beet pulp and maize silage and these plants showed a similar diversity of thermophilic microorganisms with the exception of the *Kosmotoga* genus that was only detected in Loděnice.

**Figure 6:**
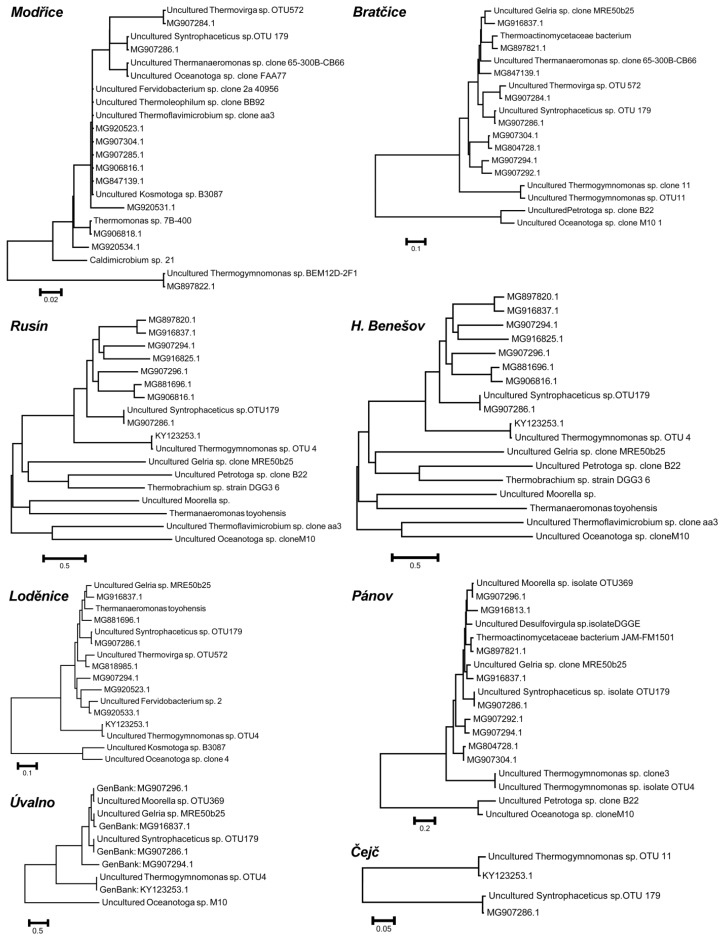
Phylogenetic trees showing thermophilic microorganisms found in the anaerobic fermenters.

Overall, *Syntrophaceticus* and *Thermogymnomonas* were the most abundant genera and were found in all anaerobic fermenters. The *Gelria* and *Oceanotoga* genera were also detected in high abundance in all fermenters with the exception of Čejč and Modřice. To clarify the genetic diversity of the thermophilic microorganisms in all these fermenters, a comparison of our 16S rRNA data was performed with GenBank and the genetic relationships are shown in phylogenetic trees (Figure 6).

## 3. Discussion

Biogas is the product of anaerobic fermentation and methane in biogas produced by methanogenic *Archaea* in the following pathways: reduction of carbon dioxide, dismutation of methanol or methylamines and fermentation of acetate [20]. Communities, which produce methane, are very resilient and stable, though they create largely undefined consortia. The aforementioned pathways can be realized by syntrophic acetate-oxidizing bacteria that convert acetate to hydrogen and carbon dioxide and simultaneously reduce carbon dioxide to methane by hydrogen-utilizing methanogens [20]. This process was described in thermophilic fermenters [21], mesophilic fermenters, [22] and natural environments [23,24].

Our research demonstrates that syntrophic acetate-oxidizing bacterium *Syntrophaceticus* sp. were the most widespread thermophilic microorganisms in all fermenters. This is probably caused by high ammonia levels leading to syntrophic acetate oxidation, a process that takes place in mesophilic fermenters [22]. The novel species *Syntrophaceticus schinkii* was discovered and isolated from sludge and from a mesophilic methanogenic fermenter operating at high ammonium concentrations [25,26]. *Syntrophaceticus schinkii* is a strictly anaerobic, mesophilic, syntrophic acetate oxidizing, spore-forming and gram-variable, bacterium with a growth temperature ranging from 25 to 40 °C. *Syntrophaceticus schinkii* is able to oxidize acetate and produce methane during cultivation with hydrogenotrophic methanogens [25]. Another dominant genus detected in each fermenter was *Thermogymnomonas*. Itoh et al. (2007) isolated *Thermogymnomonas acidicola* and this strain was described as a thermoacidophilic, cell wall-less archaeon with variable cell size and a growth temperature range of 38–68 °C (optimum 60 °C) and at pH value range 1.8–4.0 (optimum pH 3.0) [26]. This microorganism is in contrast with others we identified as it is an obligatory aerobic archeon. This genus is very often described in association with anaerobic fermentation especially when hydrolysis of cellulose occurs [27].

The other microorganisms that were also highly abundant in fermenters were from genera *Gelria* and *Oceanotoga*. One of these microorganisms *Gelria glutamica* was for the first time isolated and characterized from a propionate-oxidizing methanogenic enrichment culture (note that its habitat could be methanogenic granular sludge). *Gelria glutamica* is a strict anaerobic, moderately thermophilic, spore-forming, obligately syntrophic, glutamate-degrading, bacterium that can grow between 37 °C and 60 °C with an optimum range from 50 °C to 55 °C and an optimum pH of 7. It can growth in cultures containing glutamate, proline, and casamino acids with the hydrogenotrophic methanogen *Methanobacterium thermautotrophicum*. Glutamate is transformed to H_2_, CO_2_, propionate and traces of succinate but sulphate, sulphite, thiosulphate, nitrate, or fumarate cannot be utilized as electron acceptors [28]. The *Oceanotoga* genus was found in offshore oil-production well head at Bombay High (Western India). For example, the novel *Oceanotoga teriensis* is a thermophilic, chemo-organotrophic bacterium which growths at a range between 25 and 70 °C, with temperature optima ranging from 55 to 58 °C. One of the Bacteria in this genus, *Oceanotoga teriensis,* utilizes various carbohydrates or complex proteinaceous substances and converts them to H_2_, CO_2_ and reduces thiosulfate and elemental sulfur to hydrogen sulfide [29].

Diversity of methanogenic microorganisms and their biogas production depends on the presence of other bacteria in bioreactors, including sulfate-reducing bacterial populations [11,12,30,31]. These bacteria also use organic compounds and consequently produce toxic hydrogen sulfide [32,33,34,35,36,37]. This competition and production of hydrogen sulfide in high concentration can inhibit methanogenic *Archaea* and acetogenic microorganisms. One of the solutions to limit this inhibition could be the use of different compounds that can impede the growth of this bacterial group and their sulfate reduction [38,39,40,41,42].

The microorganisms identified in the anaerobic digesters were compared with sequences from GenBank and the resulting phylogenetic trees are shown in Figure 6. The abundance and diversity of thermophilic microorganisms depend on the composition of the substrate in each fermenter. The highest microbial variation in the distribution (11 genera) and number (40.8%) was found in the fermenter at a wastewater treatment plant. Their presence in the mesophilic anaerobic fermenters may come from the silage, where those conditions could be more than 50 °C. In this study, we identified thermophilic microorganisms in mesophilic anaerobic fermenters but it still remains unknown how physiologically or metabolically active these microorganisms were. This is a prospect for further research as here we focused on the fundamental foundations for other hypotheses and research.

## 4. Materials and Methods

### 4.1. Diversity of Biogas Plants

The biogas fermenters were located in Úvalno, Horní Benešov, Čejč, Pánov, Modřice, Rusín, Loděnice, and Bratčice in the Czech Republic. The types of substrates are presented as the ratio of fresh input substrate (w/w%). The compositions were as follows: Úvalno: maize silage, sugar beet pulp, whole crop silage, cattle manure (44/44/6/6); Horní Benešov: maize silage, sugar beet pulp, whole crop silage, cattle manure, grass silage (29/39/12/15/5); Čejč: maize silage and liquid pig manure (80/20), Pánov: maize silage, poultry litter (92/8); Modřice: primary sludge, biological sludge (50/50), Rusín: maize silage, sugar beet pulp (70/30); Loděnice: maize silage, sugar beet pulp (75/25); Bratčice: maize silage, whole crop silage, poultry litter (63/31/6). The investigated scale anaerobic digesters is presented in Table 1.

### 4.2. Sampling and Analytical Methods

Three samples were collected from each biogas plant reactor with volumes ranging from 2500 to 3500 m^3^ and operated at 40 ± 4 °C. Organic load rate was 3.5–5.5 kg org. mass/m^3^ per fermenter and feed intervals were 80–100 kg/kWhel. The samples were collected directly from the fermenter into sterile vessels. After collection, they were stored in thermocontainer and immediately transported to the laboratory for further analysis.

The temperature, volatile solids content, total solids (TS) content, pH, redox potential, and biogas composition in each anaerobic digester of biogas plant was determined. TS was determined as an amount of material remaining after the water in the sample has been evaporated at 105 °C ± 5 °C in a drying oven EcoCELL 111 (BMT Medical Technology Ltd., Brno, the Czech Republic), according to Czech Standard Method (CSN EN 14346 2007) [43]. Volatile solids content (VS) was determined as an amount of material remaining after the combustion of the samples at 550 °C ± 5 °C according to Czech Standard Method (CSN EN 15169, 2007) [44]. Muffle furnace LMH 11/12 (LAC, Ltd., Rajhrad, The Czech Republic) was used. For pH and redox potential measurement pH/Cond meter 3320 (WTW GmbH, Dinslaken, Germany) was used, in accordance with standard (CSN EN 12176, 1999) [45]. Biogas composition was estimated by the gas analyzer Dräger X-am 7000 (Dräger Safety AG&Co. KGaA, Lübeck, Germany).

The results were analyzed and plots were built using software package Origin7.0 (www.origin-lab.com). Using the experimental data, the basic statistical parameters (M–mean, SE–standard error, M ± SE) have been calculated. The accurate approximation was when *p* ≤ 0.05 [46].

### 4.3. DNA Isolation, Amplification, and Sequencing

The isolation of DNA was done by the QIAamp Fast DNA Stool Mini Kit (QIAGEN GmbH, Hilden, Germany). The sample (100 mg) was washed with 1.4 mL of ASL buffer (QIAGEN GmbH, Hilden, Germany) and it was heated at 95 °C for 10 min. For amplification of the V3 and V4 variable regions of the 16S rRNA gene fragments universal primers were used [47]. The primers were marked by molecular barcodes for sample identification. Maxima™ Probe qPCR Master Mix (Thermo Fisher Scientific, Waltham, MA, USA), was used for PCR reaction. All manipulations of amplification and sequencing were carried out as described in previous paper [12,48]. Based on the microorganisms’ presence, the calculation of relative abundance of the taxonomic groups was done. Sequences analysis was done by NCBI and by BLAST [49]. The genomic sequences are available in GenBank, access No.: MG916813.1, MG847139.1, MG906816.1, MG907296.1, MG920534.1, MG897820.1, MG920523.1, MG920523.1, MG907286.1, MG920533.1, MG907285.1, MG920531.1, MG916837.1, MG907284.1, MG907294.1, MG881696.1, MG818985.1, MG907292.1, MG916825.1, MG906818.1, KY123356.1, MG897821.1, MG907304. The sequences were processed by Geneious 7.1.9 and genomic analysis was performed [50]. Alignments of sequences were done by MEGA7 using Clustal W with the BLOSUM cost matrix, and clustered by the neighbor-joining method [51]. The results were processed and analyzed using Origin 7.0 (www.origin-lab.com).

## 5. Conclusions

Thermophilic microorganisms were characterized from various biogas plant fermenters and their diversity and abundance were determined under the effect of various input substrates and operating conditions. The presence of the different thermophiles is connected to the substrate profiles of the biogas plants investigated which may be due to an extended range of temperature response for these thermophiles. The study is providing important information considering thermophiles and methanogens that can help to better optimize biogas production. In addition, we highlight the impact of different input substrates and their influence on the diversity and the abundance of microorganisms present. Taken as a whole, this study gives a broader and clearer picture of the processes occurring in mesophilic biogas reactors in the presence of thermophiles.

## Figures and Tables

**Table 1:** The investigated scale anaerobic digesters.

Fermenter	Installed Power (kW_el_)	Fermenter Volume (m^3^)	Process Tempe-rature (°C)	Hydraulic Retention time	Daily Biogas production Rate (L_biogas_·L_ferm.vol._^−1^)	CH_4_ Content in Biogas (%_vol_) *	pH in Fermenter (−) *	Solids Content in Fermenter (%) *	Volatile Solids Content in Fermenter (%) *
Modřice	1000	6 × 3000	34	22	0.64	47	7.02	5.09	59.13
Bratčice	750	2 × 1040 1 × 1040	40	86	2.77	51.5	8.3	10.16	75.23
Pánov	500	2 × 1320 1 × 1630	41	85	1.76	48	8.03	10.33	79.46
Úvalno	750	2 × 1040 1 × 1040	40	78	2.77	49	7.69	8.84	78.85
Horní Benešov	750	2 × 1040 1 × 1040	41	85	2.77	52	7.85	7.87	77.52
Rusín	750	2 × 1970 1 × 1630	41	85	1.56	48	7.63	8.52	79.15
Loděnice	840	3 × 1970	41	90	1.64	50.5	7.65	7.9	78.51
Čejč	750	2 × 3500 1 × 3800	40	65	0.81	50.3	7.54	4.3	78.98

* Long term average.
